# REM refines and rescues memory representations: a new theory

**DOI:** 10.1093/sleepadvances/zpaf004

**Published:** 2025-01-22

**Authors:** Alessandra E Shuster, Allison Morehouse, Elizabeth A McDevitt, Pin-Chun Chen, Lauren N Whitehurst, Jing Zhang, Negin Sattari, Tracy Uzoigwe, Ali Ekhlasi, Denise Cai, Katherine Simon, Niels Niethard, Sara C Mednick

**Affiliations:** Department of Cognitive Sciences, University of California, Irvine, Irvine, CA, USA; Department of Cognitive Sciences, University of California, Irvine, Irvine, CA, USA; Princeton Neuroscience Institute, Princeton University, Princeton, NJ, USA; Department of Experimental Psychology, Oxford University, Oxford, UK; Department of Psychology, University of Kentucky, Lexington, KY, USA; Department of Psychiatry, Massachusetts General Hospital, Harvard Medical School, Boston, MA, USA; Department of Psychiatry, Athinoula A. Martinos Center for Biomedical Imaging, Charlestown, MA, USA; Department of Psychiatry and Human Behavior, University of California, Irvine, Irvine, CA, USA; Department of Cognitive Sciences, University of California, Irvine, Irvine, CA, USA; Department of Cognitive Sciences, University of California, Irvine, Irvine, CA, USA; Department of Neuroscience, Friedman Brain Institute, Icahn School of Medicine at Mount Sinai, New York, NY, USA; Department of Pediatrics, School of Medicine, University of California, Irvine, Irvine, CA, USA; Pulmonology Department, Children’s Hospital of Orange County, Orange, CA, USA; Institute of Medical Psychology and Behavioral Neurobiology, University of Tübingen Tübingen, Germany; Department of Cognitive Sciences, University of California, Irvine, Irvine, CA, USA

**Keywords:** REM sleep, learning and memory, autonomic nervous system, cognitive function, cognitive development, dreams, functions of REM sleep, NREM-REM cycles

## Abstract

Despite extensive evidence on the roles of nonrapid eye movement (NREM) and REM sleep in memory processing, a comprehensive model that integrates their complementary functions remains elusive due to a lack of mechanistic understanding of REM’s role in offline memory processing. We present the REM Refining and Rescuing (RnR) Hypothesis, which posits that the principal function of REM sleep is to increase the signal-to-noise ratio within and across memory representations. As such, REM sleep selectively enhances essential nodes within a memory representation while inhibiting the majority (*Refine*). Additionally, REM sleep modulates weak and strong memory representations so they fall within a similar range of recallability (*Rescue*). Across multiple NREM-REM cycles, tuning functions of individual memory traces get sharpened, allowing for integration of shared features across representations. We hypothesize that REM sleep’s unique cellular, neuromodulatory, and electrophysiological milieu, marked by greater inhibition and a mixed autonomic state of both sympathetic and parasympathetic activity, underpins these processes. The RnR Hypothesis offers a unified framework that explains diverse behavioral and neural outcomes associated with REM sleep, paving the way for future research and a more comprehensive model of sleep-dependent cognitive functions.

**Statement of Significance**

Since its identification in the 1950s, rapid eye movement (REM) sleep has both fascinated and puzzled researchers. Despite its omnipresence across species, significant developmental trajectory, and involvement in a variety of cognitive processes, the precise function of REM sleep has remained elusive. Here, we propose the REM Refining and Rescuing (RnR) Hypothesis as a new framework to understand the function of REM sleep in memory processing. This hypothesis posits that REM sleep serves two principal functions: refining memory representations by honing them down to their essential elements and rescuing weak memories that would otherwise be forgotten. We propose that the dual actions of refining and rescuing memory representations during REM sleep can explain a broad range of cognitive outcomes.

**Opening Remarks by Dr. Sara C. Mednick**

*Proverb:* My grandmother taught my mother who taught me and I taught my daughter who will teach my granddaughter who will teach my great granddaughter and when you add up all the generations you have almost 10 000 years of wisdom.

*Robert Stickgold: An American Scientist*

By the time I sat down to hear a guest lecture by Robert “Bob” Stickgold for an undergraduate Cognitive Science class in the basement of William James Hall, I had already gotten kicked out of a lab in the first semester of my first year of graduate school and had found safe harbor in the Harvard Vision Lab under the supervision of Ken Nakayama and Patrick Cavanaugh. Despite the emotional bruises, I felt extremely lucky to have been given a second chance and hungry to discover the research question that “keeps you up at night,” as Ken called it. I found my answer in Bob’s lecture, and in Bob himself. It was 1997, before Tetris and hypnogogic dreaming, before Science and Nature Neuroscience, before Jerry Siegel and Alan Alda.

Bob seemed to come out of nowhere with a sparkling, innovative, and creative approach to sleep research that gave this somewhat sleepy field a full make-over. He offered a deceivingly simple experimental method and ingenious analytical approach that could be adapted to answer so many new, up-at-night questions. These were the burning questions we didn’t even know we should be asking, but once they were presented in that small lecture hall, and later, on the world stage, we recognized their groundbreaking nature and indomitably inspiring potential. And his work and gifts and insights have made good on their promises and continue to lead us to the most exciting discoveries about the sleeping brain and body since the discovery of REM sleep itself.

The strangest and most compelling (for me) part of Bob’s coming up story is that he was, in true American tradition, an outsider to the field where he made his greatest marks. Trained as a bench scientist in biochemistry working in Stephen Kuffler’s lab and others, Bob’s energetic wandering mind drove him away from the predictableness of the laboratory, towards experimenting with science fiction writing (completing two books) and jobs in the “real world.” Finally, he returned to science in his fifties, but to an unknown terrain, several unknown terrains truthfully, as he inimitably married disparate and new-to-him research fields, sleep and cognitive neuroscience, and demonstrated how they fit like a lock and key.

I have felt like a science outsider myself, with a BA in drama/dance at Bard College, and my first attempt at the Ivory Tower met with a kick to the curb. For me, Bob was someone I could understand and respect, and even adore, as many of us who have been lucky enough to work with him do. This **Special Issue Festschrift in honor of Dr. Robert Stickgold** was put together by those people. As that grateful first year who eventually became Bob’s first graduate student, co-mentored by Ken Nakayama PhD, it is an honor to contribute a paper from my University of California, Irvine Sleep and Cognition Lab, which, as a group, is proud to be part of Bob’s legacy.

## Introduction

When Robert Stickgold entered the sleep field in the mid-1990s, it was abuzz with excitement about the function of rapid eye movement (REM) sleep [1–4]. However, since its identification in the 1950s [5], REM sleep has generally confounded its observers. On the one hand, it shows omnipresent conservation across most species [6], has a consistent and functionally significant trajectory across early development [7], makes up 20% of a night of sleep in humans [8], and contributes to a host of cognitive processes [9–19]. On the other hand, studies have also shown that humans may be able to survive without REM sleep, as seen in studies that block REM sleep experimentally with monoamine oxidase inhibitors and genetically in some families [20–22]. The precise role of REM sleep for cognitive function has been difficult to pin down due to uneven replication of prior findings [23–25] and similar benefits from other parts of sleep. For example, although emotional, but not neutral, memory improvement has been associated with REM sleep and REM theta power [26], similar results have been reported with nonrapid eye movement (NREM) sleep spindles [27, 28].

Perhaps most disconcerting, until recently, behavioral effects of REM sleep had not been successfully linked with specific events in human scalp electroencephalogram (EEG) that have homologous signatures in animal models. Without, for example, discrete, spatiotemporally specific burst events tied to human cognitive function that show identical sleep and cognitive outcomes in animal models, researchers have not had the building blocks to develop mechanistic, testable models, and thus, the story of REM foundered. In contrast, REM’s sibling sleep state, NREM sleep, benefitted tremendously from the identification of several EEG markers (i.e. spindles, slow oscillations [SO], sharp wave ripples) that appeared to be key players in offline memory consolidation in both human and animal models and could be algorithmically detected and experimentally targeted using a wide range of interventions (e.g, electrical, sound, smell, targeted memory reactivation [TMR]). Knowledge gained from this rush of experimental studies focused on NREM sleep has informed the principles of important theoretical models of sleep-dependent memory improvement (e.g. Active Systems Consolidation, Synaptic Homeostasis Hypothesis, Opportunistic Consolidation Hypothesis, etc.) [29–32], which explain only half the story of sleep.

We are now at the beginning of the next wave of exploration of the role of REM sleep in cognitive and brain function made possible by several factors. First, technological advances in imaging approaches in neuroscience have provided insights into REM-dependent neural function, including measurement of dendritic spines and tracking specific molecules throughout the memory process. Second, the accumulation of more research has evinced greater symmetry between human and animal research approaches and their outcomes, including magnetic resonance spectroscopy of the balance of excitatory/inhibitory neurotransmitter concentrations (E/I balance) and intracranial recordings and targeted stimulation of specific memory and neural events during sleep. Third, the recent discovery of neural events in human scalp EEG associated with cognitive changes, i.e. theta and alpha bursts during REM sleep associated with visual perceptual learning and hippocampal-dependent forgetting [33], holds promise for establishing spatiotemporal biomarkers of REM sleep processes. Building off these discoveries, we propose the *REM Refine and Rescue (RnR) Hypothesis*, which aims to explain the function of REM sleep as it contributes to how we process memories and learn from prior experiences.

As we will argue, due to its unique cellular, neuromodulatory, and electrophysiological milieu, we propose that the two principle functions of REM sleep are to *refine* and *rescue* memory representations that have been recently encoded during wake and subsequently reactivated during NREM sleep. As the word “refine” connotes a process through which unwanted elements are removed such that the final product is purified and honed, we propose REM sleep refines individual memories by stripping them down to their essential network nodes. It does so by heightening the activity of a few key neurons selectively tuned towards prioritized (i.e. learned or trained) information, while at the same time, reducing the overall activity of the majority of neurons activated by exposure to the experience at encoding. We define rescue as recovery from a catastrophic end, such as with memory that is otherwise fated for forgetting. Across all recently encoded experiences, REM sleep rescues memories by leveling up the peaks of the representations such that both strong and weak memories are more equally retrievable, similar to the digital audio process of normalizing loud and soft sounds (i.e. peak normalization). Thus, *REM’s prevailing action is to boost the signal-to-noise ratio by narrowing (refining) and normalizing (rescuing) representations.*

When considered through this lens, the RnR Hypothesis can explain many seemingly disparate behavioral findings associated with REM sleep. In this paper, we hope to demonstrate how the tenets of the hypothesis can explain: (1) synaptic development of the infant brain, (2) sharpened tuning functions of orientation-selective cells to trained visual targets, (3) prioritization of emotional over neutral memories, (4) forgetting of hippocampal memories, (5) reduction in emotional/autonomic reactions to negative stimuli, and (6) enhanced generalization and insight [34–40]. We will also show how RnR is consistent with neural data showing that REM sleep shifts the cortical excitation-inhibition balance towards reduced excitation and stronger inhibition, further lowering noise levels beyond what is seen in NREM sleep and wake and that this inhibitory shift facilitates memory [41, 42]. Conceptualizing REM sleep as an inhibitory state may seem counterintuitive to the reader given that REM sleep is classically thought of as a highly active, hyper-associative state [7, 43]. Indeed, the older term “paradoxical sleep” in reference to REM sleep was coined due to the fact that the EEG activity during this stage was similar to that of wake, suggesting a wake-like brain. However, recent findings provide evidence that cortical activity during REM sleep is shifting towards increased inhibition [41, 42, 44, 45], suggesting that the old story of REM sleep is ready for an update.

Our working metaphor aligns with the concept described by the sculptor Michelangelo. He claimed that upon beginning to work on a sculpture, he could already see the final product in the raw piece of marble and that his job was to chip away at the rock until the object lurking within could be revealed. “The sculpture is already complete within the marble block, before I start my work. It is already there. I just have to chisel away the superfluous material.” Bridging this metaphor to learning and memory, we think the process of sleep is to turn the raw, unadulterated memory network activated during encoding, i.e. the whole block of marble, into an enduring, long-term memory, i.e. the final sculpture.

Memories can be considered on many levels, from cellular to systems, and can be given many names, from engrams to representations. The term engram has been described as a memory trace coding information about an experience [35, 46, 47]. The engram is a moving target, as only a subset of cells active during encoding get co-activated during subsequent retrieval [48, 49]. As there are differing methods for defining and quantifying engrams [46, 48, 50], going forward we use the term *memory representation* to denote the ensemble of units (e.g. neurons, dendrites, synapses, etc.) that forms a specific memory that endures across initial encoding, offline consolidation, and later retrieval. Similarly, as we map out the tenets of the Refine and Rescue Hypothesis the reader may note a certain vagueness in our discussion of how the tenets apply to all levels of processing: systems to cellular, cortical to subcortical, explicit to implicit. This is intentional and stems from the dominant focus on cortical processes in the field and the small number of animals studies devoted to REM sleep. Occam’s razor would predict that physiological states (e.g. REM sleep) affect all levels of an organism similarly. Thus, we hypothesize that these tenets are relevant to all levels of processing. We hope that the testable predictions and potential mechanisms put forward will lead to more research on the curious topic of REM sleep.

### Tenets of the Refine and Rescue Hypothesis

After encoding, the first step in the offline consolidation process of a memory representation occurs during quiet rest or NREM sleep by the reactivation of the memory or partial reactivation [51]. NREM sleep also recruits high levels of nonspecific noise reduction, otherwise known as synaptic downscaling. This is then followed by REM sleep, which, we hypothesize, does the detailed work of chiseling away superfluous, experience-specific material, as well as leveling activity across all representations. And depending on the cognitive domain and the number of NREM/REM cycles, this RnR process will produce a range of parallel outcomes.

**Figure 1.**
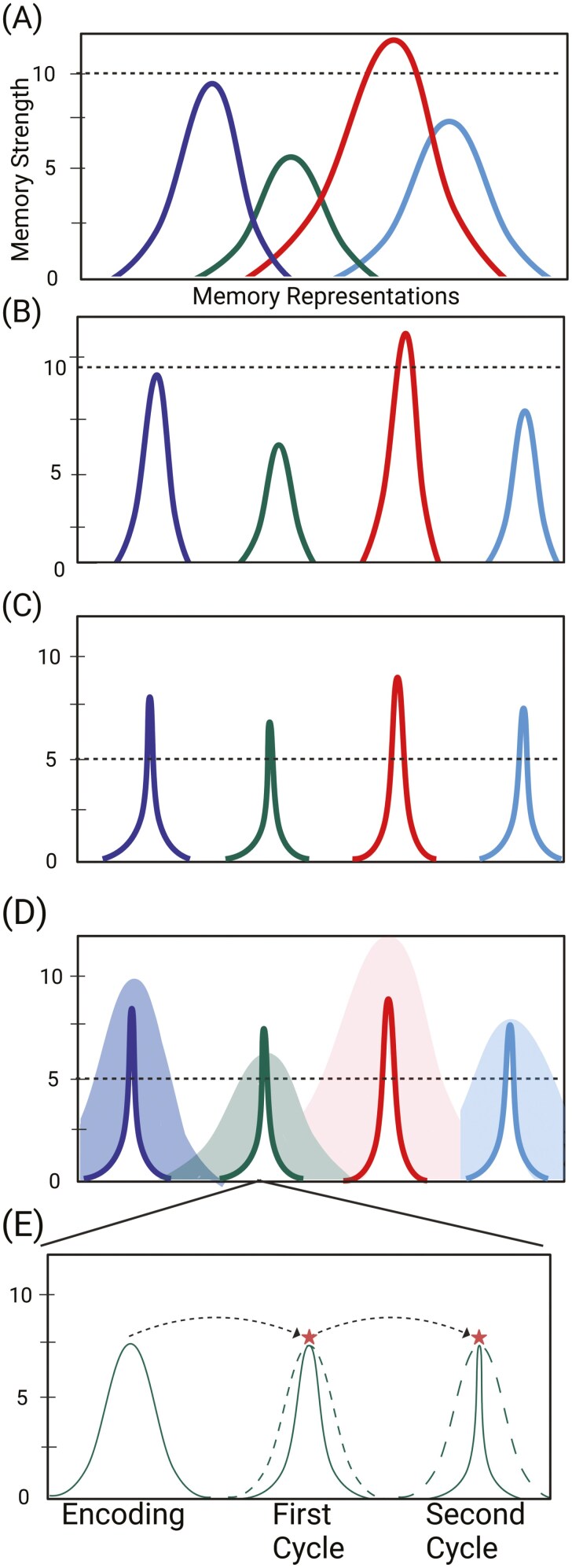
Memory representations visualized across encoding, NREM sleep, REM sleep, and retrieval. (A) Waking experience (encoding) stimulates widespread excitation of cells that form the initial memory representation. Each representation, visualized as separately colored lines along the *x*-axis, is diffuse and overlaps with other representations. The *y*-axis indicates memory strength, implicating a greater number of units (e.g. neurons, dendrites, synapses, etc.) recruited for stronger memory representations. Dashed line for *y*-axis reference. (B) During subsequent NREM sleep, the brain undergoes global synaptic downscaling, lowering overall noise of the system represented by a significant loss of excitability with each memory network. Also, memories get reactivated (during ripples nested in spindles) with encoding strength predicting amount of reactivation. The result is a profile of separable memory distributions with variable peaks of excitability, high peaks reflecting salient, strong memories, and low peaks reflecting weak memories. (C) REM sleep provides an overall inhibitory state from the synaptic to systems levels, where the inhibition of individual units, and thus the memory representation as a whole, is scaled by the magnitude of prior excitability during wake and NREM. This scaling results in the leveling of excitability across memory representations whereby both weak and strong memories are more equally represented (i.e. peak normalization), a.k.a. *Rescue.* Note that the strong and weak memories are brought within a similar range on *y*-axis of Memory Strength. Within each memory representation, REM sleep selectively inhibits excitability of most of the connections, while heightening a minority of learning-specific nodes (*Refine*). The result is a distribution of distinct memory representations that, with reduced overlap, visualizing the idea that fewer neurons are now activated by multiple memories resulting in more distinct and refined memory traces. (D) Memory representations at retrieval (dark lines) are compared with encoding representations (shaded areas) show that the offline memory consolidation process across NREM and REM sleep significantly reduces memory representations to their most essential nature and allows weak and strong memories to be more equally retrieved. (E) Representations get further refined and rescued over multiple NREM-REM cycles, heightening excitability of a minority of learning-specific nodes. Across cycles, this process may allow for peak-to-peak connections and integration across multiple representations promoting generalization, rule abstraction, and creativity.

The REM Refining and Rescuing Hypothesis posits that the principal function of REM sleep is to increase the signal-to-noise ratio within a network and proposes the following tenets:

1. Waking experience stimulates widespread excitation of multiple, overlapping memory representations (Figure 1, A, Wake).
2. During subsequent NREM sleep, the brain undergoes global synaptic downscaling along with reactivation of memory representations, resulting in a profile of individuated representations of varying strengths (peaks), where high and low peaks reflecting strong and weak memories (Figure 1, B, NREM).
3. After reactivation of recent memories during NREM sleep, REM sleep’s sui generis milieu provides an overall inhibitory state from the synaptic to systems level, where the inhibition is scaled by the magnitude of prior excitability during wake and NREM sleep. This scaling results in the leveling of excitability across memory representations whereby both weak and strong memories are more equally represented (i.e. peak normalization) (Figure 1, C, REM sleep, note that the strong and weak memories are brought within a similar range on *y*-axis of Memory Strength). We refer to this as the Rescue process.
4. For individual memory representations, REM sleep selectively inhibits excitability of most of the network, while heightening a minority of learning-specific nodes (or neurons) (Figure 1, C, REM, note the slimming of tuning functions to a narrow band of memory-specific cells). We refer to this as the Refine process.
5. Over multiple NREM-REM cycles, memory representations get further refined to their most essential peaks, which allows for peak-to-peak connections and integration across multiple representations (Figure 1, E, note how refining reduces memories to their essential natures (i.e. peaks), which can then make linkages across representations potentially promoting generalization, rule abstraction, and creativity).

In the following sections, we provide support for the RnR Hypothesis based on known behavioral correlates of REM sleep and emerging research about REM mechanisms that support these functional outcomes. We begin by reviewing the key processes of NREM and REM sleep that occur in the brain and body. Next, we review evidence of REM’s role in development and brain maturation. Following this, we find connections between the major behavioral outcomes of REM sleep across cognitive domains (e.g. perceptual learning, generalization/rule abstraction and creativity, emotional memory processing, and episodic forgetting) that can be understood in context of the tenets of the RnR Hypothesis via enhancing the signal-to-noise ratio of a memory. We additionally discuss supporting neural mechanisms that are unique to REM sleep. Finally, we summarize the ideas of the RnR Hypothesis and discuss testable predictions and future directions.

### Overview of sleep

Over a night of sleep, the human brain cycles through two primary phases: NREM and REM sleep. NREM sleep is further divided into stages 1, 2, and 3 (or slow-wave sleep) [8]. Stage 1 sleep is a transitional state from wake to sleep, making up 3% of adult nocturnal sleep. About 60% of adult sleep is stage 2 sleep, which is marked by distinct EEG events called sleep spindles and K-complexes. Stage 3, also known as N3 or slow-wave sleep, makes up about 20% of sleep, and it is marked by slow, high-amplitude oscillations called SO (<1 Hz), slow-wave (0.5–2 Hz) and delta (1–4 Hz) activity. REM sleep marks a change from NREM sleep reflected in the brain and body. Making up 20%–25% of a night’s sleep, the brain in REM sleep almost resembles that of wake and is characterized by low amplitude mixed frequency waves [8], and low-amplitude alpha (8–12 Hz) and theta (4–8 Hz) oscillations, as well as recently characterized alpha and theta bursts [33]. Additionally, during REM sleep, the body experiences muscle atonia as well as REMs [7, 52]. REM sleep is also the stage where most reported dreaming occurs [53]. A full sleep cycle consisting of NREM followed by REM sleep lasts approximately 90 min, but each cycle is not similarly composed. Sleep at the beginning of the night consists of longer chunks of slow-wave sleep, which get shorter in subsequent cycles, and short time periods of REM sleep lasting around 10 minutes, which increase to around 60 min near the end of the night [8, 54]. In total, sleep across the night consists of approximately 75%–80% of NREM and 20%–25% of REM sleep [8].

Though REM sleep comprises up to a quarter of total sleep, its mechanisms are disproportionately less understood than those of NREM sleep, though a multitude of behavioral results that will be described in the following sections show a role for REM sleep across numerous cognitive domains. Still lacking is a theoretical framework that encompasses these behavioral results in context of REM sleep mechanisms, which we attempt to address with the REM Refining Hypothesis.

## REM in Early Development

Compared with research in adults, REM sleep in early development is understudied, which is problematic given that all animals sleep the most when they are young, with REM sleep predominating during gestation and infancy compared to all other stages of life across all species [7, 55, 56]. Evidence links the quantity and quality of early REM sleep to long-term developmental and cognitive outcomes. REMs in preterm infants, which tend to occur during phasic REM sleep and couple with theta bursts in the hippocampus, have been related to better mental development index scores, suggesting that infants with more REMs during REM sleep may experience greater endogenous stimulation of the brain areas necessary for better long-term cognitive development [57–59]. Further, the total amount of REM sleep and the rate at which the proportion of REM sleep declines is associated with long-term cognitive development, including mental development index scores, motor skills, and intellectual outcomes [60, 61].

In humans, sleep states dynamically change across the gestational and postnatal period. Neural electrophysiological activity can be detected around 20 weeks gestation, with increasing organization and continuity in electrical activity. By approximately 28 weeks gestation, REM sleep, NREM, and an indeterminate sleep stage can be observed [62]. The sleep cycle becomes more structured and consistent with fetal age and parallels anatomical and neural circuit maturation [63]. At term (approximately 40 weeks), newborns’ sleep is comprised of equal amounts of REM and NREM sleep [64]. REM sleep during these early stages of life has been strongly associated with sculpting the brain through activation of developing neural circuitry and elimination of superfluous neural connections. In the neonatal stage, the brain is in a dynamic state creating new connections, both local and long-range. Endogenous neural firing and the sensory-driven activation via fetal twitches increases activation of developing neural networks [65, 66]. In his REM Sleep Ontogenetic Hypothesis, Roffwarg capitalized on this activation and theorized that REM sleep was essential for shaping early brain development through the reinforcement of neural circuitry required for conscious waking activities [67]. Consistent with this neural plasticity hypothesis, studies monitoring the sleep of preterm infants have shown that greater initial amount of REM sleep is linked to greater morphological and functional connectivity maturation [68–70].

Postnatal brain maturation is also characterized by enhanced neural connectivity, including synaptic growth, while simultaneously refining networks through selective pruning, with REM sleep implicated [71]. Using monocular deprivation techniques in kittens, early work by Hubel and Wiesel demonstrated that development of typical brain circuitry in the visual cortex was experience-dependent [72]. During this critical period, depriving one eye of visual input while allowing the other to see resulted in much narrower ocular columns in the cortical area corresponding to the deprived eye. In addition, neurons in the visual cortex that usually responded to input from both eyes, ceased responding to inputs from the deprived eye [37]. Exploiting the same technique, Frank, Issa, and Stryker demonstrated that sleep greatly enhanced cortical plasticity of ocular dominance after monocular deprivation, whereas sleep deprivation completely prevented this enhancement [73]. In this landmark study, different roles for NREM and REM sleep in plasticity began to emerge. First, changes in ocular dominance in the monocular deprivation groups positively correlated with the amount of NREM sleep in the dark period, suggesting that NREM sleep enabled the strengthening of experience-dependent changes in cortical circuits. Second, although the authors found significant increases in REM sleep following the deprivation period, REM sleep *negatively* correlated with ocular dominance plasticity. The authors commented that this surprising result suggested “a possible inhibitory effect of REM sleep on ocular dominance plasticity” [73]. Building on this work, more recent studies have confirmed the role of REM sleep in regulating developmental plasticity processes by supporting neural connectivity of large-scale networks, pruning the majority of new synapses, and strengthening the small fraction of enduring learning-related spines [69, 74]. This selective pruning and strengthening of spines occur via dendritic calcium spikes specific to REM sleep, suggesting that endogenous neuronal activity during REM sleep is critical for brain development.

Twitches, a strong feature of active sleep (the infant analog to REM sleep) postnatally, are spontaneously generated and result in widespread neural network activation [15]. Induced by the brainstem, these twitches evoke bursts of activity, similar and potentially linked with sleep spindles, that guide and refine motor networks [15, 75, 76]. Although the mechanisms and consequences of twitches have not been worked out, an area of developmental robotics has reported that mimicking the production of, and sensory feedback from, “twitches” can transform initially undifferentiated neural circuits into differentiated circuits comprising functional sensorimotor inhibitory and excitatory connections (reviewed in Blumberg et al.). It is not yet known, however, how twitch-induced activation of inhibitory and excitatory connections may drive or participate in the selective neuronal growth and refinement of REM sleep. Together, these studies implicate REM sleep as an opportune time for enhancing and fine-tuning new neuronal connectivity. In the next section, we investigate the role of REM sleep in the refinement of adult perceptual plasticity processes.

## REM and Perceptual Learning

In adulthood, nonhippocampal perceptual learning requires REM sleep. Expanding on the early sensory development studies that demonstrated an inhibitory role of REM sleep in plasticity of the visual cortex, studies have demonstrated that REM sleep fine-tunes experience-dependent sensory learning in adult humans and animals. It does so by increasing the signal-to-noise ratio of cell populations towards a trained set of visual features. In perceptual learning tasks, subjects are trained across sessions to distinguish simple features (e.g. visual oriented lines or moving dots) amongst perceptual distractors. Due to the sensitivity of early sensory neurons to simple perceptual features, learning is specific to the trained task, such that performance gains usually do not generalize to untrained stimuli such as the retinotopic location of the target and target specific features, including orientation, frequency, motion direction, and more [77–80]. One of the most robust and flexible effects of REM sleep on perceptual learning has been demonstrated with the texture discrimination task (TDT), a visual perceptual learning task. Stickgold and others have reported that improvement: (1) is specific to the trained location (retinotopic-specificity) and orientation of the target (e.g. learning does not transfer to new target locations or to untrained orientations of background elements); (2) requires six to eight hours of nighttime sleep; and (3) depends on the combined product of overnight NREM and REM sleep implicating a sequential process of learning requiring both NREM and REM sleep [11, 81–84].

As a graduate student with Stickgold, Mednick demonstrated that a well-positioned daytime nap containing both NREM and REM sleep produced the same magnitude of perceptual learning as a full night of sleep [12]. The nap paradigm had several methodological assets compared with overnight sleep, including better circadian and waking controls and experimental control of sleep stages by varying the duration of the nap (e.g. 60 min naps contained NREM-only, 90 min naps had NREM + REM). This allowed researchers to dissociate cognitive processes supported by either NREM sleep alone or both NREM and REM sleep. These studies demonstrated that (1) without a nap, repeated, within-day perceptual training fatigues visual performance in a training-specific manner, (2) naps with NREM sleep reduce training-specific fatigue, and as a result performance remains stable with repeated testing compared to deteriorating performance across wake, (3) REM sleep is required to see performance gains over baseline, with the magnitude of perceptual learning correlated with the product of NREM and REM sleep [12].

Understanding underlying mechanisms of these REM sleep-driven perceptual benefits has come from rodent studies demonstrating that REM sleep facilitates the sharpening of tuning functions of orientation-selective cells in early visual cortex for trained visual features [85, 86]. After perceptual training, sleep facilitates orientation-selective response potentiation in V1 neurons reflecting increased firing to trained orientations, a process driven by thalamocortical long-term potentiation [85, 86]. Importantly, studies show that orientation-specific information is relayed from thalamus to cortex during poststimulus sleep via sparsely firing V1 neurons that are “weakly coupled to V1 population activity,” and thus are “soloists” rather than “choirists” [85]. These soloists are more visually responsive than other V1 neurons, have greater orientation selectivity than neighboring neurons, and show firing increases across sleep. Critically, this selective increase in firing rates of soloist neurons to the trained orientation occurs preferentially across periods of REM sleep. Aton and colleagues propose that REM sleep increases activity of sparsely firing neurons specifically, which leads to differential experience-dependent plasticity (i.e. the sharpening of tuning functions of early visual neurons toward trained task features). Thus, the RnR Hypothesis would predict that REM sleep’s refining process silences the majority of cells within the choir, while simultaneously rescuing the sparsely firing neurons that respond selectively to trained visual inputs. This REM-specific process stabilizes the memory against competing stimuli, such as interference.

Perceptual learning is vulnerable to interference when competing tasks are trained in short temporal succession and share competing stimulus features (e.g. same spatial location) [87–90]. When examining visual perceptual learning and interference, researchers determined that NREM sigma activity (12–15 Hz) correlated with local plasticity in trained target areas of visual cortex and over-sleep performance gains. Subsequent REM sleep demonstrated a complementary role that stabilized visual memory and facilitated resistance to interference, with REM theta activity in occipital regions important for stabilization [39]. Using magnetic resonance spectroscopy to measure glutamate and GABA concentrations in early visual areas, subsequent work demonstrated differences in the excitatory/inhibitory balance from NREM to REM sleep [45]. They reported that GABA decreased during NREM sleep, which *increased* overall E/I balance, regardless of whether learning had occurred prior to sleep or not. In contrast, glutamate decreased during REM sleep, which *lowered* overall E/I balance. Importantly, this decrease in E/I balance was only found in the learning condition, not in the nonlearning control condition. Moreover, they found that these differences in E/I balance in NREM and REM sleep may mechanistically support the stabilization of visual memories. Before the final retrieval test, participants were tested on a version of the TDT that has previously been shown to cause interference. They found that whereas the increase to E/I balance during NREM positively predicted sleep-dependent performance gains, the reduction of E/I balance during REM sleep was associated with resilience to interference. These findings suggest that visual memories get re-instantiated during NREM sleep, and during REM sleep, a time of reduced excitation, training-specific enhancement in signal-to-noise occurs. Thus, REM is recruited to refine away “superfluous” aspects of the memory representation and rescue and stabilize essential nodes, making them resistant to future interference.

One of the strongest pieces of evidence of REM sleep’s peak normalization of weak and strong memories comes from studies reporting that REM sleep rescues memories that have already experienced interference. McDevitt et al. trained participants on three separate texture discrimination displays yielding three different levels of task-based interference—high retroactive interference, moderate proactive interference, and low interference. Following the interference induction, participants either remained awake (active wake or quiet wake), took a nap with NREM sleep only, or took a nap with both NREM and REM sleep. Findings indicated that for the condition with high retroactive interference, performance was completely disrupted when the interference learning was followed by active wake, quiet wake, or NREM-only sleep. However, performance completely recovered when interference learning was followed by a nap with both NREM and REM sleep, implicating that REM sleep specifically was critical for rescuing these memories [17]. The authors speculated that during REM sleep, the competing task representations were separately tuned (perhaps due to increased activity of the sparsely firing “soloists”), thereby decreasing neural overlap and rescuing behavioral performance, an idea consistent with the peak normalization of weak and strong memories such that they are equally available at retrieval. Interestingly, in this study, the low and moderate interference conditions did not depend on REM sleep to yield good performance, which suggests that when learning conditions are sufficient to establish relatively distinct representations, REM sleep tuning mechanisms may not elicit detectable behavioral effects different from wake or NREM-only. Future work is needed to understand how selectivity for refining and rescuing occurs during REM sleep, e.g. how does the brain select information to be remembered versus to be forgotten.

The idea of fine-tuning representations is also related to the finding that REM sleep improved recognition of novel objects in deep camouflage [91]. In this task, training involved viewing novel objects embedded in different camouflaged backgrounds. To successfully segment a novel object from the camouflaged background without any external cues, the visual system must learn from the image statistics to group continuities for segmentation of object boundaries [92]. Findings showed that REM sleep facilitated the extraction of novel objects embedded in many different backgrounds, suggesting that the fine-tuning necessary for new learning may occur by abstracting across many exposures to eventually arrive at a visual insight. Considered in the framework of RnR, refining object representations to their essential features by amplifying the signal coding the trained object and decreasing the background noise and then repeating this process across multiple exposures and multiple cycles of NREM and REM sleep, leads to the extraction and integration of shared image statistics of an object or, even, of a concept (Figure 1, E).

Together, these results suggest distinct and complementary functions of NREM and REM such that: (1) NREM is a state of learning-independent plasticity that activates entire perceptual memory representations leading to nonspecific performance gains. (2) REM sleep’s inhibitory state of learning-dependent plasticity excites sparsely firing, training-selective cells while reducing overall background activity, resulting in refined individual representations that are more resilient to interference, and where weak and strong representations are relatively equated across the memory landscape. The next question is how do these sharpened tuning functions act over time across multiple iterations of NREM and REM sleep. For the answer, we turn to a growing literature on the benefits of REM sleep for generalization and creativity.

## REM and Generalization, Rule Abstraction, and Creativity

The ability to formulate a generalized concept by extracting commonalities across multiple experiences is foundational for human reasoning and an important part of the creative process. Several studies have demonstrated a role for REM sleep in extracting generalizations and rules, as well as making creative connections [14, 36, 93, 94]. In this section, we review these studies and elucidate how the mechanisms of the REM RnR hypothesis can lead to these outcomes via repeated iterations of enhancing signal over noise that highlights connections between the central nodes of each network.

Researchers have used TMR to show that cueing a memory during REM sleep may facilitate generalization and rule abstraction. Sterpenich et al. associated emotional and neutral faces with sounds and then played the sounds during subsequent NREM and REM sleep. Cueing during REM sleep was associated with enhanced retrieval memory for encoded faces, but also for faces not seen at encoding (i.e. false alarms), suggesting that REM-cueing facilitated generalization to face categories. Also, cueing during REM sleep, compared to NREM sleep and wake, favored corticocortical reactivations and led to the formation of novel auditory-visual associations at retrieval, measured using functional magnetic resonance imaging. Together, these results suggest that REM sleep benefits the creation of new associations, as well as integration of new experiences with semantically related knowledge representations. Repeated exposure to a category of stimuli (e.g. faces) may refine each representation to its essential features and, over multiple NREM-REM sleep cycles, allow for the extraction and integration of shared features. Additionally, forming links across multiple representations may lead to the generation of novel cortical representations reflecting generalizations or an overarching set of rules and concepts that can be applied to new experiences.

Supporting these ideas, results from Schapiro et al. using a novel semantic category learning task, demonstrated that the extraction of shared features within a semantic category was dependent on sleep, compared with wake. Critically, time in REM sleep was associated with better extraction of the shared features specifically for the low frequency, weaker features, consistent with a process that refines representations to their essential nodes while also peak normalizing weak and strong representations. Intriguingly, the authors reported parallel yet stronger effects across a whole night of sleep (multiple cycles of NREM/REM sleep) compared to a nap (single cycle of NREM/REM sleep), suggesting that the shared feature extraction increased with more opportunities for reactivation during NREM sleep and refine and rescue during REM sleep [95]. Consistent with this finding, Pereira and colleagues (2023) showed that TMR cueing during REM sleep, but not NREM sleep, benefitted rule abstraction, but the cueing benefit in the REM sleep group did not emerge until one-week post-TMR manipulation [94]. This extended time-frame implicates an iterative process of many cycles of NREM/REM sleep in the emergence of rule abstraction. Thus, we believe that REM sleep plays a critical role in the process of generalizing episodic experiences to overarching semantic categories and abstracting an underlying grammar common to a set of related experiences. REM sleep’s signal-to-noise enhancement of representations eventually builds to forming connections between the central nodes across memory representations. How this spreading activation across refined representations occurs within the brain needs to be elucidated, but the evidence suggests that this process may lead to the discovery of associations between disparate, weakly associated ideas, which is a fundamental component of creativity.

Early work by Stickgold demonstrated a role for REM sleep in creative thinking by demonstrating that performance in solving anagrams was enhanced and that participants were better in learning weak (e.g. thief–wrong) but not strong primes (e.g. hot–cold) after awakening from REM sleep, compared with NREM sleep [43, 96]. Here, weak primes are reflected by remote associations that are often used as an index for creativity. In a similar vein, Cai et al. demonstrated that REM sleep uniquely supports the ability to leverage associative links primed before sleep into useful postsleep creative problems [14]. Compared with NREM-only naps and wake, only naps with REM sleep facilitated the utilization of primed words in solving creative problems on a remote associates test, evincing a 40% improvement in creativity. If we agree that implicit priming of words elicits weaker memory representations, compared with explicit encoding, this result is consistent with the concept that REM sleep levels up weaker memories with other representations, facilitating their use in solving creative problems. A follow-up study replicated REM sleep’s boost of weak primes for use in a creativity task and demonstrated the significant mechanistic role of the autonomic nervous system (ANS) during REM in this process [36] (see *REM Autonomic Activity Supporting Cognitive Processes* section). In summary, we propose that REM RnR increases the brain’s ability to uplift faint glimmers of thoughts and experiences, consistent with peak normalization, making them more available to be incorporated into novel and useful ideas (a process important for creativity [97]) and the formation of abstract concepts).

## REM and Emotional Memory

Further evidence of REM sleep’s function in enhancing signal-to-noise comes from studies of emotional processing and sleep. Acute emotional experiences have been shown to follow a common trajectory, entailing an early phase in which emotions dominate over reason, followed by a gradual reduction of emotional reactivity and increase in cognitive strategies used to process the event [98, 99]. Accordingly, emotional experiences are thought to be composed of both an autobiographical, episodic component and an emotional component [98, 100]. This transformation of emotional experience across time has been shown to be facilitated by sleep, specifically REM sleep [26, 101–104]. We review the two principal outcomes of sleep-dependent emotional processing that are consistent with the RnR Hypothesis, whereby, in the early phase, REM sleep enhances signal-to-noise of a memory by heightening activity of salient emotional nodes within a memory representation and reducing activity of neutral, episodic details, that are more weakly connected within the representation, relative to the emotional aspects (refining). Additionally, over time REM sleep supports changes in the memory representation (rescuing) with a shift in emphasis from heightened emotional reactivity to greater episodic long-term memory of the experience [105]. Together with NREM’s strengthening of episodic aspects of the memory, REM sleep promotes healthy emotional processing, leading some researchers to consider REM sleep as overnight therapy [106].

Within 24 h of being exposed to both emotionally salient and neutral experiences, humans preferentially remember the emotionally charged details at the expense of the neutral aspects, an effect termed the emotional memory trade-off effect [34, 107]. Over time one’s emotional reactivity (perceived valence and arousal) to the stimulus decreases, while aspects of the episodic component are maintained. The Sleep to Forget Sleep to Remember hypothesis posits that REM sleep is an optimal brain state for these changes to occur due to coordinated reactivation of encoding-related regions during REM sleep during hippocampal theta oscillations, as well as decreased amplitude of affective tone due to decreased aminergic activities [108]. Low central adrenergic activity during postencoding REM sleep has been associated with lower amygdala activity, decreased emotional reactivity to emotional items, and greater prefrontal connectivity after sleep [108]. Importantly, the emotional memory trade-off is frequently caused by the forgetting of neutral memories, while the emotional memories are preserved [40, 109, 110]. Groch had participants encode negative and neutral pictures with frames around each image and then compared memory for the images and frames after NREM-rich or REM-rich sleep. The emotional memory trade-off was found only after REM sleep, with reduced memory for neutral stimuli and maintenance of the emotional stimuli. On the other hand, after NREM sleep greater recall was shown for the color of the frames associated with neutral memories. Other work has demonstrated a causal role of NREM sleep spindles in this process [27]. Thus, NREM spindle activity may facilitate the reactivation of a wide range of information associated with the memory, whereas REM sleep may refine the representation to its essential node(s) by facilitating forgetting of superfluous, neutral details.

**Figure 2.**
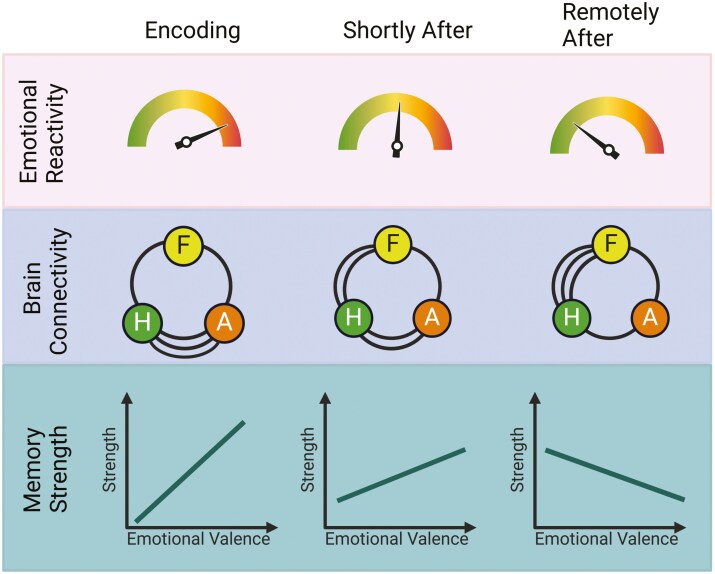
REM sleep and emotional memory. This figure illustrates how three components of an emotional experience (emotional reactivity, brain connectivity, and memory strength) change across time. Time is depicted on the *x*-axis, including Encoding, Shortly After (Encoding), and Remotely After (Encoding), with greater time from encoding indicating more cycles of REM sleep. The top panel depicts the spectrum of emotional reactivity, from low (green) to high (red). The middle panel shows changes in connectivity between the Hippocampus (H), Amygdala (A), and the Frontal Cortex (F), with the number of lines indicating the magnitude of connectivity. The bottom panel depicts changes in the relation between memory strength (*y*-axis) and emotional valence associated with a memory (*x*-axis, neutral to more emotional values from left to right). Similar to Figure 1, greater memory strength (*y*-axis) signifies greater number of units (e.g. neurons, dendrites, synapses, etc.) recruited. Considering a real-life, negative experience, such as a robbery at gunpoint, at the moment of the robbery, emotional reactivity heightens, hippocampal-amygdala connections engage with encoding the experience, which biases memory strength towards emotional details of the experience (i.e. the weapon is remembered more than more neutral details, such as the shirt color of the assailant). Shortly after the event, the victim of the robbery has had at least one night with REM sleep and emotional reactivity diminishes, brain connectivity begins to shift toward hippocampal-frontal connections, and the relation between memory strength and emotional valence shows less bias towards emotionally salient details. Remotely after the emotional experience, including many nights of REM sleep, recall of the event triggers less reactivity, with greater connectivity between the hippocampus and frontal cortex and pruning of amygdala connections (e.g. refine). Memory strength is now biased towards originally weaker, neutral details (i.e. rescue) over emotionally salient components.

Emotional memory representations shift over time. Where at first recall may be saturated by heightened emotional reactivity, eventually our memory of these fraught or thrilling events shifts to emphasize episodic details at the expense of the feelings aroused by the memory. This transformation may also be supported by REM sleep. Werner et al. exposed participants to negative and neutral images and then gave them either a nap with or without REM sleep [111]. Subjects rated the aversiveness of the images directly after the nap conditions, as well as intrusions from the negative images on three subsequent nights. Critically, REM sleep during the nap was *positively* associated with postnap aversiveness ratings and *negatively* associated with intrusion ratings three nights later. These results suggest that after an emotional experience, REM sleep shapes the trajectory of an emotional memory from emphasis on emotional reactivity in the short-term to de-emphasis in the long-term. Several studies have shown similar results, with REM theta activity implicated [26]. Consistent with this idea, REM sleep has been implicated in the extinction of strong fear memories [112–114]. Viewed through the lens of RnR, this process signifies a peak shift within an emotional memory representation where memory strength shifts from being associated with emotionally charged details to episodic details that start out as weaker memories (relative to the emotional details) and eventually become stronger. Through the peak normalization process, over time REM sleep rescues weaker aspects within the emotional memory by inhibiting amygdala-related emotional connections and strengthening prefrontal-related episodic connections (Figure 2) [108, 111, 112, 115].

Growing evidence demonstrates a role for dreaming in the transformation of emotional memories across time (with ANS activity during REM sleep and dreaming discussed in: REM Autonomic Activity Supports Cognitive Function). According to the emotion regulation theory of dreaming, dreams provide a safe space to process and regulate our emotions, particularly negative emotions. In one seminal study, depressed divorcees who dreamed about their ex-spouses were more likely to have significantly reduced depressive symptoms 1-year later [116]. In addition, dreams recalled with more detailed memories and emotions tended to be associated with decreased depressive symptoms at follow-up, implicating a therapeutic role for dreaming [117]. In another study that induced stress prior to sleep, participants who dreamed about the stressful event had a more positive attitude towards the experiment the next morning than those who did not dream about the event, demonstrating a potentially adaptive role of dreaming [118]. Similarly, having dreams with negative content has been associated with greater adaptive emotion regulation the following day [93]. We hypothesize that the autonomic arousal that occurs when one dreams about an emotional experience may be a critical part of the transformation of both emotional reactivity and episodic memory aspects of that experience.

Accordingly, along with reductions in emotional reactivity, recent findings show that dreams may be involved in the emotional memory trade-off [40]. In this study, participants were exposed to emotional and neutral images, followed by assessments of their memory and emotional reactivity to these images before and after sleep. Upon waking the next morning, participants provided detailed descriptions and rated the emotional intensity and valence of their dreams. Results indicated that only participants who reported dreaming exhibited the emotional memory trade-off effect over sleep, i.e. maintaining negative memories while forgetting neutral items, alongside reduced emotional reactivity postsleep. Furthermore, the emotionality of the dreams significantly influenced reactivity to previously viewed negative images, with more positive dreams leading to greater reduction in next-day reactivity to neutral pictures. In this study, it is not clear whether the recalled dreams occurred during REM sleep, but the morning dream report procedure likely increased REM sleep dream recall. Furthermore, REM sleep is the stage where dreams are recalled more frequently, and mental activities recalled from REM sleep tend to be more vivid, bizarre, and dream-like [119]. These findings together implicate an important role of dreaming in REM sleep’s unique effects on memory, an area that requires further investigation.

In summary, the primary outcomes of the emotional memory process across sleep involve the emotional memory trade-off (e.g. prioritization of emotional over neutral memories) and the gradual dissipation of emotional reactivity and a shift toward greater memory for less emotional, episodic details over time. We hypothesize that REM sleep facilitates both processes by refining acute emotional experiences (signal) while forgetting neutral details (noise) in the short-term, and rescuing episodic information (signal) over emotional tone (noise) within memory representations in the long-term. We have reviewed evidence that dreams may support these processes, implicating their mechanistic role in the RnR processes and identifying dreams as potentially therapeutic targets for interventions in emotional disorders.

### REM Autonomic Activity Supports Cognitive Function

A small but compelling body of research suggests that the ANS may play a role in NREM and REM-related processing of cognitive and emotional experiences [36, 120–125]. Here, we consider potential contributions of autonomic activity during REM sleep specifically. We will first summarize autonomic states during NREM and REM sleep and then provide links between autonomic activity during REM sleep and memory consolidation, along with providing potential mechanistic links to the RnR hypothesis.

Bidirectional communication between the ANS and the brain, specifically the central autonomic network (comprised of the locus coeruleus, hypothalamus, amygdala, and ventromedial prefrontal cortices), form a feedback loop that maintain adaptive responses to environmental changes. The central autonomic network receives peripheral input from the two branches of the ANS via sympathetic preganglionic neurons and the parasympathetic vagus nerve. NREM sleep is characterized by parasympathetic vagal dominance, including lower heart rate and blood pressure, as well as increased heart rate variability (HRV) measured in time-based approaches (e.g. root mean square of successive differences) and frequency-based approaches (e.g. high-frequency HRV [HF-HRV; 0.15–0.40 Hz; ms^2^]). In contrast, REM sleep engages sympathetic activity, including increased heart rate and low-frequency HRV (LF-HRV; 0.04–0.15 Hz; ms^2^), and a higher LF/HF ratio (LF[ms^2^]/HF[ms^2^]), an indicator of sympathovagal balance [126–130]. Interestingly, despite relatively greater sympathetic dominance during REM sleep, the absolute activity of the parasympathetic branch (HF-HRV) is equivalent during REM and NREM sleep [121, 123]. Additionally, during REM sleep, there is a notable increase in acetylcholine, the primary neurotransmitter of the parasympathetic nervous system, which increases inhibitory activity during REM sleep, while locus coeruleus-norepinephrine activity is typically silent [131, 132]. During REM sleep, amygdala activity assumes greater control over cardiovascular regulation and HRV compared with wake and NREM [133]. Thus, similar to wakefulness, REM combines high levels of both sympathetic and parasympathetic activities [134], yet potentially via different mechanisms. Intriguingly, this mixed autonomic state may vary depending on phasic and tonic REM, as sympathetic activity during REM sleep is particularly increased during phasic REM [135, 136] and tends to coincide with delta activity (0.05–4 Hz), theta bursts in the hippocampus, and REMs, which are separately associated with increased amygdala activity [137–139]. Furthermore, age-related hyperactivity of locus coeruleus during wake is associated with disturbed REM sleep, specifically reduced REM theta activity [140]. Taken together, despite the limited research in the functional role of brain-heart interactions during REM, it’s distinct blend of sympathetic and parasympathetic activity coupled with phasic REM, theta bursts, and increased amygdala regulation hints at a potentially important role for autonomic activity in REM-related memory processing.

The mixed autonomic state during REM sleep may play a role in the refining and rescuing actions of REM, as parasympathetic activity during REM sleep has been associated with rescuing weak memories, boosting weakly primed targets, and enhancing weaker neutral memories over salient emotional ones in healthy and clinical populations [36, 114, 141]. Using a creativity task in which targets were explicitly (strong memory) or implicitly (weak memory) trained before naps with and without REM sleep, Whitehurst and colleagues demonstrated that only naps with REM sleep improved performance in the priming condition. Importantly, high parasympathetic activity during REM sleep was the strongest predictor in strengthening accessibility of primed (weak) targets for later creative thinking, accounting for over 75% of the variance in performance improvement [36]. In a different context, Morehouse et al. examined the role of parasympathetic activity during REM sleep in the emotional memory trade-off effect, in which emotional memories are prioritized over neutral memories. Using a placebo-controlled, within-subjects design, they examined the impact of suppressing HF-HRV with zolpidem (Ambien), a nonbenzodiazepine GABA-agonist, on overnight memory change for negative and neutral images. In the placebo condition, higher HF-HRV during REM sleep predicted greater memory for neutral images at the expense of negative images. On the other hand, when HF-HRV was suppressed in the zolpidem condition, participants recalled more negative than neutral memories, suggesting that greater parasympathetic activity during REM sleep reduced dominance of the strong negative memories, and enhanced consolidation of the neutral, arguable weaker, memories [141]. Extending these findings to a clinical population, another study examined the role of REM parasympathetic activity in emotional memory processing and vulnerability to posttraumatic stress disorder (PTSD). The researchers exposed participants with PTSD to a fear conditioning task followed by extinction learning, which required participants to override a salient fear memory with a neutral, weaker memory. After extinction learning, participants slept (monitored by EEG and ECG), and then were retested on the extinction memories upon waking. PTSD patients with disrupted REM sleep, specifically poor parasympathetic activity during REM, showed an inability to learn the extinction memory [114]. Taken together, these three examples of an association between REM parasympathetic activity and rescuing weak memories are consistent with the peak normalization process, further implicating parasympathetic activity during REM sleep as playing a mechanistic role in the RnR model, an intriguing and testable concept with clinical implications.

**Figure 3.**
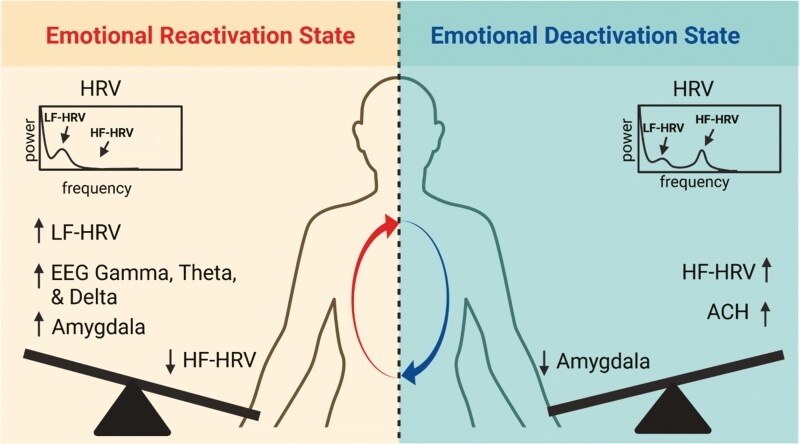
Autonomic activity during REM sleep and emotional memory. Cyclical autonomic states in REM sleep: A sympathetic-dominant emotional Reactivation state and a parasympathetic-dominant emotional Deactivation state. We propose that the reactivation of emotional memories during phasic REM sleep activates the sympathetic-dominant emotional Reactivation state, where LF-HRV activity is increased and tends to coincide with delta activity (0.05–4 Hz), theta bursts in the hippocampus, REMs, and amygdala regulation. Dreaming may also be involved in this state, with bursts of sympathetic activity, which are linked to frontocentral gamma, signaling the reactivation of emotional components of a memory. In response to this Reactivation state, descending projections from the central autonomic network send top-down signals to the ANS that engage the parasympathetic vagal “break” leading to a reduction in sympathetic activity and an emotional Deactivation state. We propose that this state is characterized by increased HF-HRV and acetylcholine (ACH), the primary neurotransmitter of the parasympathetic nervous system, which increases inhibitory activity during REM sleep. During the Deactivation state, phasic REM amygdala activity is reduced. We hypothesize that the Reactivation-Deactivation feedback loop during REM sleep, interleaved with NREM sleep, may inform the trajectory of emotional and cognitive processing over time.

A potentially important key to understanding REM sleep’s role in emotional memory processing is the engagement of sympathetic and parasympathetic activity during dreams. As we have mentioned, a leading consideration of dream function is to provide a safe space for processing negative experiences and dreaming has been associated with better emotional outcomes. Mechanistically, dreaming involves reciprocal communication between the brain and body, with one study showing highly dynamic interplay between sympathetic arousal and frontocentral gamma-band EEG activity during dreams [142] although other studies have shown alternate results regarding REM gamma [108, 143]. What is not known is whether the therapeutic benefit of dreams is associated with the unique brain-body communication during dreams. While speculative, we suggest that bursts of sympathetic activity during dreams signal a “reactivation” state where emotional components of a memory are coupled with increased arousal response in the limbic system (e.g. amygdala, cingulate gyrus, hippocampus, and sympathetic arousal). In response to the arousal, the ANS naturally engages the “vagal break” through increased parasympathetic activity, which may function as a “deactivation” state allowing the dreamer to reduce emotional reactivity during the dream (Figure 3). We propose that this dynamic feedback loop of reactivation and deactivation states may be one potential mechanism underlying the postsleep reduced emotional reactivity and rescuing of episodic details over time. Dreams provide an optimal state for this ANS feedback loop wherein fraught dreams of an emotional memory may lead to greater long-term downscaling of reactivity.

In summary, while only a handful of studies exist on the relation between autonomic activity during REM sleep and cognitive outcomes, we think the literature points to the exciting possibility that the intricate balance of sympathetic and parasympathetic activity during REM sleep may be a sophisticated mechanism where memory processes are dynamically regulated. Sympathetic arousal during dreams may trigger the reactivation of emotionally significant memories (refine), while parasympathetic activity may foster better emotional regulation and reduction of the strength of negative memories along with enhancement of neutral episodic details (rescue, peak normalization). Prior work has proposed a Slow Oscillation Switch Model [121], which posits that NREM sleep naturally toggles between two states: parasympathetic-dominant and spindles/ripples dominant, each of which leads to specific functional outcomes for working and long-term memory, respectively. It’s an intriguing idea that vagal parasympathetic activity is critical for both NREM processes, such as frontal cortical glymphatic clearance, synaptic downscaling, and executive function improvement, and REM processes, such as peak normalization, rescuing weak memories, and reducing emotional reactivity. We hypothesize that cyclical ANS states in REM may guide these processes. As such, autonomic processes during REM serve an essential role in fine-tuning cognitive and emotional functions, thus enabling adaptive behavior.

## REM and Forgetting

Direct evidence of REM sleep’s role in refining memory networks by inhibitory processes can be found in its role in forgetting. Several different methodological approaches have demonstrated a wide range of mechanisms specific to REM sleep that appear to facilitate forgetting. For example, Izawa et al. had mice complete two memory tasks before and after sleep: contextual fear conditioning and novel object recognition, where hippocampal-dependent activity during sleep is needed for long-term consolidation [144, 145]. They focused on hypothalamic melanin concentrating hormone (MCH) neurons that project to the hippocampus, which are implicated in sleep-wake regulation and REM sleep specifically, as MCH activation increases time in REM sleep and inhibition results in fewer transitions to the REM stage during sleep [146]. Izawa’s study found that enhancing MCH cell activity during REM sleep led to memory forgetting, while inhibiting MCH neurons yielded improved performance or less forgetting for contextual fear conditioning and novel object recognition. Inhibiting MCH neurons during wake or NREM sleep did not affect memory performance, and there was no effect of ablating MCH neurons for cued fear conditioning memory, which is thought to be more amygdala-dependent rather than hippocampal-dependent. Together, this study provides evidence that: (1) inhibitory MCH neurons facilitate sleep-dependent forgetting; (2) MCH mechanisms of forgetting are exclusive to REM sleep as manipulating MCH neurons during wake or NREM sleep did not alter memory performance; and (3) REM-active MCH selectively affects forgetting on hippocampus-related memories.

At the circuit level, dendritic spine elimination during sleep is a critical process for forgetting and memory refinement. Indeed, studies using in vivo imaging on synaptic plasticity during sleep in mice have emphasized the role of NREM sleep in the selective upregulation of memory-specific synaptic spines and the role of REM sleep in memory-specific synaptic pruning [147, 148]. Zhou et al. examined the role of sleep on dendritic spine pruning in the primary visual cortex and frontal association cortex using monocular deprivation and cued fear conditioning. Results showed that REM-deprived mice showed reduced spine elimination after monocular deprivation and fear conditioning but no differences in spine formation. Moreover, REM-deprivation resulted in more forgetting in the fear conditioning task compared with controls, implicating dendritic pruning during REM as a refining mechanism that modulates extent of forgetting [148].

In humans, physiological evidence for REM sleep-specific mechanisms that facilitate forgetting is still sparse. However, a recent study identified REM burst activity in human scalp EEG that appears associated forgetting of hippocampal-dependent episodic memories [33]. In this study, participants completed a hippocampal-dependent episodic memory task and a nonhippocampal-dependent visual perceptual learning task before and after a night of sleep. Results demonstrated that REM alpha bursts predicted overnight forgetting on the episodic memory task. Specifically, greater alpha burst power was associated with reduced word-pair memory from pre- to postsleep. Total alpha power showed a similar, but less significant, effect, whereas nonburst alpha power was not associated with sleep-dependent memory. Further investigation is needed on the relation between REM alpha bursts and other REM outcomes reviewed here (i.e. cellular mechanisms of forgetting, emotional memory trade-off, the role of dreaming, etc.) to provide the optimal context for which these REM refining mechanisms may take place.

In addition to linking alpha bursts with cognitive processes, this study also showed that theta bursts in posterior regions were associated with better performance on the TDT but not with overnight learning [33]. This result is consistent with Tamaki who reported that REM general theta power in posterior regions was associated with resilience to interference, but not sleep-dependent perceptual learning. Interestingly, Tamaki also reported that greater theta power was correlated with learning-dependent decreases to E/I balance during REM sleep [45]. Together, these studies suggest that REM theta bursts are potentially a physiological biomarker of increased inhibition and the stabilization of visual perceptual learning. Further studies should also probe which brain structures may be involved with regulating burst events, such as the medial prefrontal cortex [149], as well as examine a potential link with other REM forgetting mechanisms, such as theta phase procession [150]. Poe et al. demonstrated in rodents that hippocampal place cell neurons coding novel places are active during peaks of REM theta oscillations (associated with long-term potentiation and increased plasticity), while neurons coding familiar places are active during the trough (associated with long-term potentiation and decreased plasticity) [150]. Poe proposed that “REM sleep would serve to maintain or strengthen memories until they are transferred outside the hippocampus whereupon they should be erased from that space-limited short-term memory factory, allowing those synapses to be used to encode new associative memories.” And given the importance of forgetting in healthy emotional memory processing, REM bursts may provide a specific target for interventions to improve cognitive outcomes in relation to sleep and emotional disorders [151–153].

## REM Refining Mechanisms

What are the physiological underpinnings of the REM RnR Hypothesis? Recent studies have investigated the role of NREM and REM sleep for synaptic downregulation during sleep, reporting that global down regulation of cortical AMPA receptor expression levels is only facilitated by NREM sleep, not REM sleep [154, 155], with a specific role of Homer1a gene expression regulated by norepinephrine (also associated with regulation of sympathetic and parasympathetic activities) [156–158]. While direct structural evidence linking REM sleep to the reduction of excitatory synapses remains scarce [74, 148, 154], studies investigating network activity suggest that neocortical and hippocampal activity decreases predominantly during REM sleep [159–161]. Initially, this might seem contradictory. However, it is unclear whether this decrease is solely due to the downregulation of excitatory connections or if increased inhibition during sleep also plays a role. In fact, recent evidence in humans and rodents showed that synaptic inhibition increases during sleep while synaptic excitation decreases [44, 162], suggesting that sleep pushes the cortical network towards increased synaptic inhibition.

We additionally see that NREM and REM sleep influence inhibitory processes in the sleeping brain in distinct ways. NREM sleep spindles affect the excitability of subsets of cortical cells differentially. Additionally, pyramidal cells, especially active during spindles, increase their activity contrasting with downregulation of overall cell population activity during NREM sleep [161]. This finding supports the notion that spindles selectively enhance representation-specific excitatory synaptic connections during NREM. Consistent with the refining aspects of the RnR hypothesis, subsequent REM sleep then reduces the activity of spindle-active cells, indicating that within the same cell, both synaptic strengthening and weakening can occur during NREM and REM sleep [161]. This shows that cortical circuits undergo distinct modulations of local inhibition during NREM sleep microstates, such as SO up-states and sleep spindles, particularly when spindles occur during the SO upstate. These dynamics create unique configurations of local disinhibition that in turn promote synaptic plasticity and thereby facilitate the signal-to-noise ratio of memory representation reactivations and memory consolidation during sleep [163]. Following this, REM sleep is characterized by ongoing inhibitory activity, which subsequently shifts the cortical excitation-inhibition balance towards overall reduced excitation and stronger inhibition, lowering noise levels beyond NREM and wake [41, 42]. However, it is important to note that this does not imply REM sleep is an inactive state. Overall activity levels can remain high, as seen in measures such as multiunit activity or blood-oxygen-level-dependent signals. These measures, however, do not differentiate between shifts in excitation and inhibition.

Against the background of oscillatory activity, recent research has examined the role of nonoscillatory aperiodic EEG activity during sleep. Based on a computational model by Gao and colleagues, several studies have proposed nonoscillatory aperiodic EEG activity (typically 20–50 Hz) as a surrogate of E/I balance [164]. A steeper slope of the aperiodic power spectra is thought to correspond to greater inhibition and thus reduced overall E/I [164–166]. Recently, a study utilized in vivo calcium imaging of excitatory and inhibitory circuits with simultaneous EEG recordings during sleep in mice, as well as scalp and intracranial EEG recordings in humans, to investigate sleep-dependent recalibrations of neuronal circuits [167]. Remarkably, they discovered that REM sleep decreased aperiodic activity and that this reduction predicted subsequent memory recall. Interestingly, their calcium recordings from mice indicated that the reduction of aperiodic activity during REM sleep mainly reflected decreased excitatory activity rather than increased inhibition. This suggests that while REM sleep is accompanied by increased inhibitory activity of interneurons, the beneficial effects on memory are not an immediate result of this increased inhibition but rather depend on the successful reduction of excitatory activity caused by increased inhibition [41, 42]. Consistently, Tamaki reported learning-dependent decreases in excitatory activity during REM sleep, which contributed to lower E/I balance and stabilized visual perceptual learning [45]. Together, these pieces of evidence from both human and animal models suggest that the specificity of REM-dependent learning is related to REM sleep’s inhibitory state. However, it is important to note that the changes in E/I balance within a REM cycle are not homogenous and fluctuate [161, 168]. This fluctuation potentially separates REM sleep in substates with periods of high inhibition leading to periods of low excitatory activity, the latter being the main predictor of memory performance. Together, these demonstrate complementary processes in NREM and REM sleep, in which NREM oscillations are key for synaptic plasticity that also determine plastic processes during the next REM cycle, which serves to increase inhibition and to reduce overall excitability, consistent with the REM RnR Hypothesis as well as the behavioral outcomes we have highlighted above.

## REM Refining Hypothesis: Summary

NREM and REM sleep are functionally separate and crucially linked. We propose that after encoding, NREM sleep regulates both downscaling of overall noise in the system and reactivates partial ensembles of nodes coding for prior experiences, i.e. hippocampal-dependent memory representations and hippocampal-independent sensory or motor networks. Following NREM, REM sleep both: (1) refines individual memory representations by heightening activity of sparsely firing soloist neurons and dampening activity of majority choir neurons and (2) peak normalizes memory representations such that weaker memory representations are rescued and leveled up to the strength of strong memory representations. Across multiple NREM-REM cycles, this refinement process may allow for integration of peaks across multiple representations. While our metaphor for the first memory representation-specific process is Michaelangelo’s sculpture within the block of marble, our metaphor for how cycles of NREM and REM sleep act on multiple memories is comparable to the process of geological erosion. First, NREM sleep strengthens the relief by eroding the valleys (reduction of synaptic potentiation during NREM sleep, Figure 1, B), which leads to a relative increase in the relief. Once the relief has been sufficiently formed during NREM sleep, the erosion of the peaks can take place during REM sleep so that the heights of the elevations gradually equalize without becoming integrated into the valleys (peak normalization across strong and weak memories during REM sleep, Figure 1, C).

Considered within this framework, a wide body of research related to REM sleep can be better understood. During development, we see that REM sleep is essential for experience-dependent pruning of neural connections, which predicts cognitive outcomes and brain maturation. In adults, REM sleep is linked to perceptual learning, particularly enhancing the tuning functions of visual neurons, increasing signal-to-noise across populations of cells, and predicting specificity of behavioral outcomes. Across multiple cycles of NREM and REM, we see a benefit of REM sleep for the extraction of generalizations, abstract rules, and creative insights, with parasympathetic activity during REM sleep supporting these cognitive processes. REM sleep additionally prioritizes emotional memories at the expense of neutral memories, aiding their retention over time while diminishing the emotional reactivity associated with these memories. Dreams and the interaction between central and autonomic systems during REM sleep are potentially crucial to this process. Finally, recent research provides evidence that REM sleep supports the forgetting of hippocampal memories, with biomarkers such as EEG burst events, synaptic pruning, and MCH cell activity providing insights into this refinement mechanism.

### REM RnR and Existing Computational Models

The tenets of the REM RnR hypothesis are additionally consistent with existing computational models that use artificial neural networks to simulate neural dynamics during REM sleep. The Complementary Learning Systems framework proposed that the brain required two differentially specialized learning and memory systems, the hippocampus as a sparse, pattern-separated system for rapidly learning episodic memories, and the neocortex as a distributed, overlapping system for gradually integrating across episodes to extract latent semantic structure [169]. Postencoding, the hippocampus replays recently acquired information offline during sleep, gradually “teaching” this information to the neocortex resulting in the construction and updating of semantic knowledge over time. Norman and colleagues modeled the role of NREM sleep in the replay of recent experiences, but it additionally focused on the replay of already well-learned patterns of activity during REM sleep as a means for reduced forgetting. They proposed that a learning mechanism exists during REM sleep that allows oscillating inhibition in the network to identify weak or damaged memories so that the individual memory traces can be strengthened and repaired while also identifying and distancing memory competitors, aligning with the rescue tenet of the RnR Hypothesis [170].

Several of these models have also focused on how sleep solves the problem of catastrophic forgetting, a problem that arises when artificial neural networks overwrite previously learned information when trained on a new task [170–172]. Modeling work has shown that when new learning is interleaved with REM sleep episodes, previously learned information is preserved [170]. Researchers recently expanded this model to include hippocampo-cortical interactions during alternating NREM and REM sleep cycles, showing that during simulated NREM sleep, when the hippocampus and neocortex are tightly coupled, newly learned information became the focus of replay [172]. This functions to strengthen the representation of the new information in neocortex as well as older, related information already stored in neocortex. In their model that simulated REM sleep, the neocortex was allowed to operate with no hippocampal influence, and replay focused on repairing the old, related information to allow for “graceful continual learning.” Gonzalez et al. developed a biophysical model of thalamocortical architecture to examine how multiple competing memories can be reinstated during NREM to prevent catastrophic forgetting and that the dynamics of REM sleep could leveraged to rescue damaged memories from interference [173]. Together, these models suggest potential underlying mechanisms of REM sleep that refine memory representations and rescue weaker memories damaged by interference or age.

A recent model that closely aligns with the RnR Hypothesis developed a neural network model of acetylcholine-regulated mechanisms underlying differential NREM- and REM-specific effects on memory storage [132]. They show that reduced acetylcholine signaling during NREM sleep leads to disinhibition and excitability changes in principal cells that increases recruitment of new neurons into newly encoded memory representations. In contrast, the dramatic increase in acetylcholine during REM sleep increases activation of inhibitory interneurons that promote competitive and selective pruning of synaptic connections within memory representations [132]. This REM-dependent mechanism reduces overlap between different memory representations following a period of NREM sleep, which is crucial in real-world conditions where multiple memories are encoded and consolidated concurrently. Repeated iterations of NREM-REM sleep cycles across a night of sleep promote further expansion and segregation of memory representations in the network. These modeling results align with our RnR Hypothesis: NREM sleep reactivates swaths of information related to recent learning experiences, and then REM sleep works to reduce each memory representation to its most essential points, bringing up weak memories and reducing stronger memories. Eventually, these individual peaks in each representation can be connected to other such peaks of representations to form general patterns, rules, and insights. Reactivating related memories during REM sleep may not only serve to repair and prevent catastrophic forgetting of those memories but can also be viewed as a key to determine which aspects of the new memory should be strengthened/retained versus pruned/forgotten, a speculation that would be exciting to explore in future work.

Theories about why we dream are also consistent with the RnR hypothesis. Francis Crick’s original idea that “we dream to forget” proposed that, during REM sleep, an antilearning mechanism (e.g. reverse spike-timing-dependent plasticity) decorrelates weaker, less relevant aspects of memories, resulting in forgetting the unessential and sharpening the essential [174]. Erick Hoel proposed a similar idea, the overfitted brain hypothesis, positing that this process occurs over cycles of NREM and REM dreaming [38]. Without dream sleep, Hoel proposes that the brain experiences the danger of “overfitting,” which is the lack of generalizability that occurs in a deep neural networks when its learning is based too much on one particular dataset [38]. Dreams help mitigate this issue by injecting “corrupted inputs,” or top-down noise, to improve generalization. This suggests that the reason we dream is in response to an overfitted memory; after learning something new, dreaming adds purposeful, corrupted input (noise) to support generalization for that memory [94, 175]. Physiologically, during REM sleep the brain is in a state of both heightened sympathetic arousal, as well as the strong top-down regulation of that arousal by vagal parasympathetic inputs. A compelling suggestion is that during REM dreams, the brain toggles between two states: an excitatory state in which bursts of sympathetic arousal simultaneously with a reactivated memory adds more variance to the memory representations (Hoel’s “corrupted input”), and an inhibitory state dominated by the parasympathetic nervous system that opportunistically reduces connections to superfluous information (synapses, details, connections with amygdala and peripheral arousal). Through an iterative process with NREM and REM sleep, the brain develops the highly relevant points of a memory across many instances or reactivated patterns of activity, and the spreading activation of these highly relevant points allows for generalization. A speculative and testable hypothesis.

### Open Questions

We are now at a new wave of REM sleep research, which leaves open many exciting and unexplored territories. Along with many other ideas, we do not know enough about the neuromodulatory milieu of REM sleep and how it contributes to memory-refining processes. For example, what is the role of norepinephrine and acetylcholine across NREM and REM sleep, and how they relate to locus coeruleus and basal forebrain activity in these RnR processes? In particular, emotional and salient experiences during waking stimulate locus coeruleus and norepinephrine (LC-NE), and we see phasic LC-NE activity during NREM, which has been loosely associated with memory improvement in rodents, with no tests of emotional memory [176–178]. However, during REM sleep LC-NE activity is silent while acetylcholine is high [179]. Further research should explore the dynamics across both NREM LC-NE activity and REM ACH activity as they pertain to memory refining and rescuing processes.

We focused the scope of the RnR Hypothesis on cortical networks, as most research on REM sleep-specific circuit activity and its effects on memory consolidation has been conducted here. However, other brain areas, such subcortical structures like the amygdala and its projections, as well as REM-promoting structures in the brainstem, are well-positioned to influence these processes and warrant further investigation [180]. Finally, we have intentionally avoided making specific claims about how the RnR Hypothesis may differ in its implications for hippocampal-dependent versus cortical memories, an area that requires a multidisciplinary, -scale, -model approach.

Another frontier for sleep research is understanding the role of the ANS in REM sleep and its importance for cognitive processes. For instance, how do fluctuating parasympathetic and sympathetic states in REM sleep play a role in memory consolidation? Moreover, how does this mixed autonomic state during REM dreams shape a memory? Also, autonomic activity during REM sleep strongly predicts memory consolidation, but we have little understanding of these mechanisms. Considering the role of REM sleep in the strengthening of weak memories, which we posit is related to peak normalization across multiple memory representations, does autonomic activity play a role in this process? In light of recent results demonstrating a trade-off between autonomic activity and sleep spindles during NREM sleep that leads to a functional trade-off between working and long-term memory [121, 123], does autonomic activity support similar or different functions during REM sleep?

Additionally, it is important to determine biomarkers associated with REM refining and rescuing processes in both humans and animals and understand how these may mechanistically shape memories. For instance, do REM EEG bursts tied to episodic forgetting [33] causally shape a memory and are they related to increased inhibitory activity and decreased excitation during REM sleep? Moreover, do they relate to other features of REM sleep such as REMs, which appear to reflect gaze shifts in the virtual world of REM sleep [181]? At a larger level, can we identify mechanistic through-lines from waking experience to NREM reactivation to REM refining processes? And across time, how frequently are memory representations refined and rescued during consecutive NREM-REM cycles across nights and days? Taking a further step back, are REM RnR mechanisms in early development similar to those of adulthood, and can developmental REM mechanisms be predictive of cognitive trajectories across the lifespan?

In summary, the REM Refining and Rescuing Hypothesis underscores REM sleep’s sophisticated role in memory consolidation that is pivotal for cognitive function. The key tenets of the REM RnR Hypothesis provide a cohesive framework for understanding how REM sleep, and sleep more broadly, contribute to cognition. It additionally offers promising avenues for future research, such as exploring the role of neuromodulators and ANS dynamics during REM sleep in memory processes and identifying REM biomarkers linked to memory refinement and rescuing. Ultimately, unraveling these mechanisms will deepen our understanding of sleep’s role in learning and memory across the lifespan, informing interventions for memory- and emotion-related pathologies and enhancing cognitive performance.

## Notes

This article is part of the Festschrift in honor of Dr. Robert Stickgold Collection.
